# Parental warmth, parental rejection, and vocational outcome expectations: the mediating roles of conscientiousness and perfectionism

**DOI:** 10.3389/fpsyg.2026.1861992

**Published:** 2026-08-07

**Authors:** Filiz Gültekin, Hazel Duru

**Affiliations:** Bursa Uludag University, Nilüfer, Bursa, Türkiye

**Keywords:** conscientiousness, parental rejection, parental warmth, perfectionism, vocational outcome expectations

## Abstract

**Introduction:**

This study investigated how conscientiousness and perfectionism mediate the relationship between parental warmth and parental rejection and Vocational Outcome Expectations (VOE).

**Methods:**

Data from 729 university students (ages 17–42) were ana lyzed via structural equation modeling.

**Results:**

Results showed that perceived parental warmth (both maternal and paternal) positively predicted VOE, with conscien tiousness partially mediating these relationships. While zero-order correlations indicated a negative relationship between parental rejection and VOE, structural models revealed more complex dynamics. Specifically, perfectionism acted as an inconsistent mediator (suppression effect) in the relationship between maternal rejection and VOE, whereas perceived paternal rejection was indirectly associ ated with VOE through perfectionism.

**Discussion:**

The theoretical and practical implications of these findings are discussed.

## Introduction

University years represent a period during which individuals construct their vocational identities and make significant decisions regarding their futures. During this developmental stage, individuals typically make academic choices that will shape their careers and attempt to forecast the potential positive or negative outcomes of these choices. In this context, one of the significant cognitive variables that individuals incorporate into their career planning is vocational outcome expectations (VOE).

VOE are defined as an individual’s expectations regarding the outcomes—such as success, status, financial gain, or personal satisfaction—that they will derive from a specific occupation or academic field (Lent et al., 1994). VOE is a more specific manifestation of outcome expectations, one of the three core factors of Social Cognitive Career Theory (SCCT). When evaluated in terms of the role of occupation in an individual’s life, VOE represents the individual’s beliefs regarding the outcomes they will achieve throughout their life through their profession. Research indicates that VOE are associated with numerous career variables, including career goals (Chan, 2020), job satisfaction (Lee, 2020), job crafting (Masood et al., 2023), proactive career behaviors (Zhao et al., 2025), and turnover intention (Harb et al., 2024).

VOE is associated with numerous personal and environmental factors. For instance, a study conducted with Native American college students revealed that perceived discrimination negatively predicted students’ STEM career outcome expectations (Turner et al., 2022). Another significant factor associated with VOE is the family (Işık, 2013; Aslan and Koçak, 2025).

Within SCCT, parental support is an important contextual factor in career development (Lent et al., 1994; Lent and Brown, 2006). Parental attitudes are associated with career development (Huerta et al., 2022; Zhang et al., 2021). Particularly in collectivist cultures, career choice is motivated by the desire/need to meet parental expectations (Griffin and Hu, 2019). For example, among Asian Americans, the family has strong effects on both career choice (Hui and Lent, 2018; Fouad et al., 2008) and VOE (Shen et al., 2014). The strongest contribution to predicting VOE also stems from parental support (Işık, 2013). Rohner (1975) defined parental behaviors along a continuum of acceptance and rejection. This continuum ranges from acceptance, which includes providing love and warmth, to rejection, which extends from reprimand to hostility. The effects of childhood acceptance and rejection typically extend into adulthood and old age (Rohner, 2021). For example, acceptance/rejection is associated with educational achievement in early adulthood (Lorijn et al., 2022; Ralte and Fente, 2018). Interpersonal Acceptance-Rejection Theory (IPARTheory) posits that the way acceptance and rejection are perceived in childhood creates universal effects on personality development, social adjustment, and psychological well-being (Şirvanlı-Özen and Olgaç, 2019). Parental warmth facilitates self-efficacy and the belief that effort will be productive and success is possible (Raque-Bogdan et al., 2013). Therefore, parental acceptance or rejection can shape an individual’s personality traits. For instance, meta-analytic studies demonstrate that parental expectations and criticism influence perfectionism (Curran and Hill, 2022). On the other hand, personality traits are also associated with VOE. For example, the personality traits of conscientiousness, openness to experience, agreeableness, and neuroticism are correlated with VOE (Harb et al., 2024).

In conclusion, there is a vast literature on the interrelationships among parental influences, VOE, and personality traits. However, no research to date has examined the relationship between parental influence and VOE within the context of parental acceptance/rejection. Furthermore, this study focuses on the mediating role of conscientiousness and perfectionism personality traits in the relationship between parental acceptance/rejection and VOE. This study aims to determine the role of personal factors in the relationship between VOE and environmental factors. To this end, the mediating role of conscientiousness and perfectionism in the relationship between parental acceptance/rejection and VOE is investigated.

### Vocational outcome expectations and parental acceptance/rejection

SCCT assumes that when outcome expectations are positive, the likelihood of individuals forming intentions and exhibiting behavior increases, whereas negative outcome expectations tend to discourage individuals from performing the behavior (Lent et al., 1994). Studies demonstrating the importance of outcome expectations in developing vocational interests and directing goal-oriented behaviors support this assumption (Lent and Brown, 2019). Possessing high outcome expectations allows individuals to address challenging tasks with intrinsic motivation (Margolis and McCabe, 2006). If students believe their efforts will not lead to desired outcomes such as good grades or successful employment, they may disengage from their goals (Metheny and McWhirter, 2013). VOE represents a positive belief, and when individuals expect ideal outcomes consistent with their personal development, they are more likely to participate in career activities to achieve their goals (Vroom et al., 2015).

Prior literatüre robustly demonstrates that career decisions are significantly influenced by the environment (Savickas et al., 2009). SCCT is an approach that seeks to systematically explain the contribution of the interaction between core cognitive, personal, and environmental variables to an individual’s career (Wang et al., 2022). This approach suggests that contextual variables shape an individual’s career interests and choices (Lent et al., 1994). Among contextual variables, family and other social factors contribute to an individual’s academic and career performance at different stages. While greater social support increases engagement in career preparation, personality traits—specifically conscientiousness and extraversion—facilitate taking an active role in coping with challenges (Hirschi et al., 2011).

All human beings require love, attention, and acceptance from significant others throughout their lives (Rohner, 2021). In IPARTheory, acceptance and rejection are treated as a continuum. At one end, there is the warmth, love, care, comfort, attention, and nurturing support an individual can provide to another; at the other end, there is the absence or significant withdrawal of these positive emotions and behaviors, as well as various physically and psychologically injurious behaviors and effects (Rohner, 2021). IPARTheory studies support the assumption of a universal relationship between parental acceptance-rejection and psychological adjustment (Rohner and Britner, 2002). It has been found that family structure variables (e.g., parental occupations) and family process variables (e.g., warmth, support, cohesion, autonomy) influence many career constructs throughout the lifespan. Research shows that individuals who are loved and supported by their families achieve more satisfying outcomes in their career choices (Whiston and Keller, 2004). In a study by Aslan and Koçak (2025) with Turkish university students, it was observed that the family’s influence on career choice was a significant predictor of VOE. Vela et al. (2015), in their study with Mexican American adolescents, determined that the perception of family importance is a significant predictor of VOE. Latino students hold vocational outcome expectations such as finding work to provide for and take care of their families (Vela et al., 2019). Based on these theoretical and empirical results, we proposed the following hypotheses:

*H1*: Parental warmth (H1a maternal warmth, H1b paternal warmth) is positively associated with VOE.

*H2*: Parental rejection (H2a maternal rejection, H2b paternal rejection) is negatively associated with VOE.

### The mediating role of conscientiousness

The scope of the conscientiousness personality trait and previous studies suggest that it may play a mediating role in the relationship between parental acceptance-rejection and VOE. Conscientiousness defines the tendency to be organized, planned, careful, self-disciplined, and goal-oriented (Costa and McCrae, 1992). VOE is associated with self-transcendence, self-consciousness, and self-control skills (Sarı et al., 2017). These are traits also possessed by individuals with high conscientiousness. Conscientious students possess high intrinsic motivation (McGeown et al., 2014) and academic self-efficacy (Li et al., 2025), and consequently, achieve high academic success (Kırkağaç and Öz, 2017). These traits are also fundamental psychological foundations that support success in professional life (Wilmot and Ones, 2019). We can relate conscientiousness to Bandura's (1997) concept of “outcome expectation.” If a person believes that their efforts will lead to successful results, they work with perseverance in line with this belief. Conscientious individuals possess more of the traits necessary to work with perseverance. Within this framework, conscientiousness supports not only an individual’s behavioral consistency but also a positive expectancy system regarding the future (Lee, 2020).

There are many studies regarding the relationship between parental attitudes and personality traits (e.g., Aloia and Strutzenberg, 2022; Cuadri et al., 2025; Metwally, 2018). In line with the literature, it is believed that accepting and supportive parental attitudes foster children’s sense of responsibility. IPARTheory posits that parental rejection elicits reactions of anxiety, insecurity, low self-esteem, low self-efficacy, and emotional instability (Rohner, 2021). These reactions may not be compatible with traits such as being planned, organized, and goal-focused. Individuals who lack self-confidence and do not view themselves as worthy or competent may struggle with setting goals and exhibiting disciplined behavior toward those goals. Consequently, the following hypothesis was formed:

*H3*: Conscientiousness has a mediating role between parental warmth (H3a maternal warmth, H3b paternal warmth) and VOE.

### The mediating role of perfectionism

Conscientiousness includes traits such as planning, self-regulation, and focus on achievement. Perfectionistic individuals are defined by an excessive emphasis on order, organization, and tidiness (Frost et al., 1990). In this context, it can be said that in terms of moderation and excess, conscientiousness and perfectionism are on two separate sides regarding certain behaviors and attitudes. For example, both personality traits involve setting standards and striving to reach them. However, while one can speak of attainable goals for conscientiousness, an emphasis on high standards can be made for perfectionism. According to McCrae and Costa’s (1999) dynamic personality theory, broad personality traits play a role in the development of lower-level personality traits. Findings regarding the relationship of conscientiousness with perfectionism and its role in its development support this (Cruce et al., 2012; Stoeber et al., 2009). Therefore, while the mediating role of responsibility (conscientiousness) in the relationship between parental acceptance and VOE was examined in the study, the role of perfectionism in the relationship between parental rejection and VOE was addressed.

Perfectionism is increasingly being addressed as an important personality trait for career development. Perfectionism is associated with negative career thoughts (Andrews et al., 2014). Gnilka and Novakovic (2017) suggest that perfectionism can influence career-based outcomes in either a positive or negative way, and therefore, more comprehensive models explaining the possible mechanisms through which perfectionism can influence career outcomes should be developed. Parental expectations are among the potential causes of perfectionism (Flett and Hewitt, 2002). Perfectionistic individuals attach great importance to their parents’ expectations and evaluations (Frost et al., 1990). According to IPARTheory, acceptance leads to reactions that influence fundamental components of personality, such as a strong sense of self, confidence, and emotional balance. In parental rejection, anxiety, insecurity, hostility, low self-esteem, and cognitive distortions are observed (Rohner and Khaleque, 2010). In this context, parental rejection may lead to performance-related anxiety, low self-esteem associated with the inability to reach high standards of perfection, and cognitive distortions regarding flawless performance. This situation can also negatively affect an individual’s expectations regarding their professional life. Considering the relationship between perfectionism and VOE (Sarı, 2025; Suh and Flores, 2023), high goals and excessive sensitivity toward reaching these goals may cause individuals to have more pessimistic expectations regarding the returns they will obtain from their careers in the future. From this perspective, we proposed the following hypothesis:

*H4*: Perfectionism has a mediating role between parental rejection (H4a maternal rejection, H4b paternal rejection) and VOE.

This study aims to determine how the relationship between parental acceptance-rejection and VOE functions through personality traits. To this end, the traits of conscientiousness and perfectionism were addressed in the study. When considered in terms of parental influence on career development, the study is not novel. However, to our knowledge, previous studies have not focused on how parental influence can produce indirect effects on VOE through personality traits. Specifically, the role of a personality trait such as “conscientiousness,” which directly influences an individual’s career-related planning, effort, and persistence behaviors, has not been emphasized. Similarly, there are limited studies on the role of a personality trait such as “perfectionism,” which emphasizes high standards and extreme sensitivity regarding reaching these standards, in the relationship between these two variables.

When considered in terms of its theoretical basis, SCCT is a theory that inherently emphasizes parental support. However, in this study, a behavior that may be more injurious than mere expectations and criticism—specifically in the context of parental rejection—is addressed. Rohner (1975) states that parents can be rejecting toward their children in four different ways: cold/unloving, hostile/aggressive, indifferent/neglectful, and undifferentiated rejection. Although SCCT emphasizes the importance of environmental and contextual support for VOE, this study seeks to reveal the transformation of parental warmth or rejection in the individual’s inner world within a developmental framework, in accordance with IPARTheory. The study is structured specifically around the link between IPARTheory’s universal personality sub-theory and the individual’s core personality patterns, such as conscientiousness and perfectionism. Thus, the study attempts to present a holistic model regarding the relationship between early interpersonal relationships (IPARTheory) to future-oriented cognitive career variables (SCCT) through the individual’s personality traits. It is believed that examining this, particularly in collectivist and relational cultures such as Turkey, will contribute to the SCCT and IPARTheory literature.

Focusing on VOE during the period of emerging adulthood merits distinct attention because, at this stage of life, young adults typically complete their education and enter the workforce. This study is expected to provide significant evidence regarding how childhood family experiences shape career-related cognition and attitudes in emerging adulthood, and the long-term consequences of parent–child interaction on career development.

Although parental acceptance and rejection are theoretically conceptualized as opposite poles of the same continuum within IPARTheory, the present study modeled parental warmth and parental rejection as separate constructs to examine whether they exhibit distinct patterns of association with VOE and personality traits. Our findings suggest that these dimensions may show different pathways of influence, indicating that considering both parental warmth and rejection provides a more comprehensive understanding of their roles in young adults’ vocational development.

## Method

### Participants and procedure

A sample of undergraduate students (*N* = 729) from various departments at a large university in the Marmara Region of Turkey participated voluntarily. The sample consisted of 119 males and 610 females aged 17 to 42 (M = 21.05, SD = 2.06). Data were collected via a 20-min online self-report form including demographic items and scales assessing perceived parental acceptance/rejection, conscientiousness, perfectionism, and VOE.

### Measures

*The Adult Parental Acceptance-Rejection Scale Short Form* (*PARA/S -* Rohner, 2005). Dedeler et al. (2017) adapted the scale to Turkish. The scale form is a 4-point Likert, consists of 24 items, and has four sub-dimensions. These sub-dimensions are warmth-affection, indifference-neglect, hostility-aggression, and undifferentiated rejection. The scale is filled out separately for mothers and fathers. While a total score can be obtained from the scale, separate scores can also be obtained from each sub-dimension. The scores obtained from the warmth-affection sub-dimension of the scale indicate the level of warmth that individuals perceive from their parents. The lowest score obtained from the scale is 24, and the highest is 96. The internal consistency coefficients of the adult PARQ/S mother form were found to vary between 0.75 and 0.92, while the internal consistency coefficients of the father form varied between 0.85 and 0.96 (Dedeler et al., 2017). The reliability coefficient of the dimension used was 0.95 for the mother form and 0.95 for the father form.

*Frost Multidimensional Perfectionism Scale* (*FMPS-* Frost et al., 1990). It was adapted into Turkish by Kağan (2011). The scale consists of a total of 36 items and six subdimensions. Each item on the scale is rated on a 5-point Likert-type scale ranging from 1 (strongly disagree) to 5 (strongly agree). These subdimensions can be expressed as follows: concern about making mistakes, personal standards, parental expectations, criticism from family, uncertainty about one’s actions, and order. The Cronbach’s Alpha reliability coefficient for the entire scale was 0.91, 0.85 for concern about making mistakes, 0.79 for personal standards, 0.84 for parental expectations, 0.72 for parental criticism, 0.64 for uncertainty about one’s actions, and 0.94 for order (Kağan, 2011). This study used the parental expectations and criticism subdimensions of the scale. In the study, the Cronbach Alpha reliability coefficient was found to be 0.91 for the entire scale, 0.82 for the parental expectations sub-dimension, and 0.80 for the parental criticism sub-dimension. Although the FMPS comprises six dimensions, the present study did not aim to investigate perfectionism as a broad multidimensional personality disposition. Rather, it focused specifically on the family-related dimensions of perfectionism reflected in the Parental Expectations and Parental Criticism subscales. These subscales were selected because they most directly capture perceived parental standards and criticism, which are conceptually aligned with the study’s focus on parenting experiences and their associations with career-related outcomes. Nevertheless, because these dimensions are conceptually related to parenting experiences, discriminant validity between parental rejection and perfectionism was explicitly evaluated. Discriminant validity was established according to the Fornell and Larcker (1981) criterion. Specifically, the square root of the AVE for the Perfectionism construct exceeded its correlations with Parental Rejection in both the maternal model (√AVE = 0.81 for Perfectionism; √AVE = 0.84 for Maternal Rejection) and the paternal model (√AVE = 0.81 for Perfectionism; √AVE = 0.85 for Paternal Rejection), indicating that the two constructs were empirically distinct despite their conceptual relatedness.

*Five-Factor Personality Traits Scale* (*BFCI-* Benet-Martínez and John, 1998). BFCI is a 5-point Likert-type, 44-item measurement tool. The scale consists of five factors: extraversion, emotional instability (neuroticism), agreeableness, conscientiousness, and openness to experience. The scale was adapted to Turkish by Sümer and Sümer (2005), and the reliability coefficients for the subdimensions varied between 0.64 and 0.77. In this study, the conscientiousness subscale of the instrument was used, and its Cronbach’s alpha reliability coefficient is 0.72.

*Vocational Outcome Expectation (VOE) Scale* (McWhirter et al., 2000). The scale, consisting of 12 items and one dimension, was adapted into Turkish by Işık (2010). Higher scores on the scale indicate higher VOE. The Cronbach alpha internal consistency coefficient of the scale, which was scored as a four-point Likert scale (e.g., “My career plan will lead to a result that will satisfy me.”), was calculated as 0.87 (Işık, 2010). The Cronbach alpha reliability coefficient calculated in this study is 0.91.

### Statistical analyses

Prior to the main analyses, data screening and cleaning procedures were rigorously conducted to ensure data quality and meet the assumptions of structural equation modeling (SEM). Initially, a total of 757 responses were recorded. First, incomplete questionnaires (with more than 10% missing data, *n* = 6), duplicate submissions (identified via unique timestamp/IP screening, *n* = 2), and cases showing careless or invalid response patterns were removed. Careless responding was screened by detecting straight-line responders (participants who selected identical options across entire scales, yielding zero variance on non-reversed items, *n* = 8). Next, to ensure the assumptions of multivariate normality were met, univariate and multivariate outliers (extreme values) were systematically examined. Univariate outliers were screened using standardized z-scores (*n* = 7), while multivariate outliers were identified using Mahalanobis distance (*n* = 5) based on a chi-square cutoff criterion (critical chi-square = 45.31, *df* = 20, *p* < 0.001) (*n* = 5). After removing all invalid, outlier, and extreme cases (*n* = 28), the final analytical sample consisted of 729 participants. The final dataset was screened for missing values and outliers, and was considered suitable for SEM analysis.

**Figure 1:**
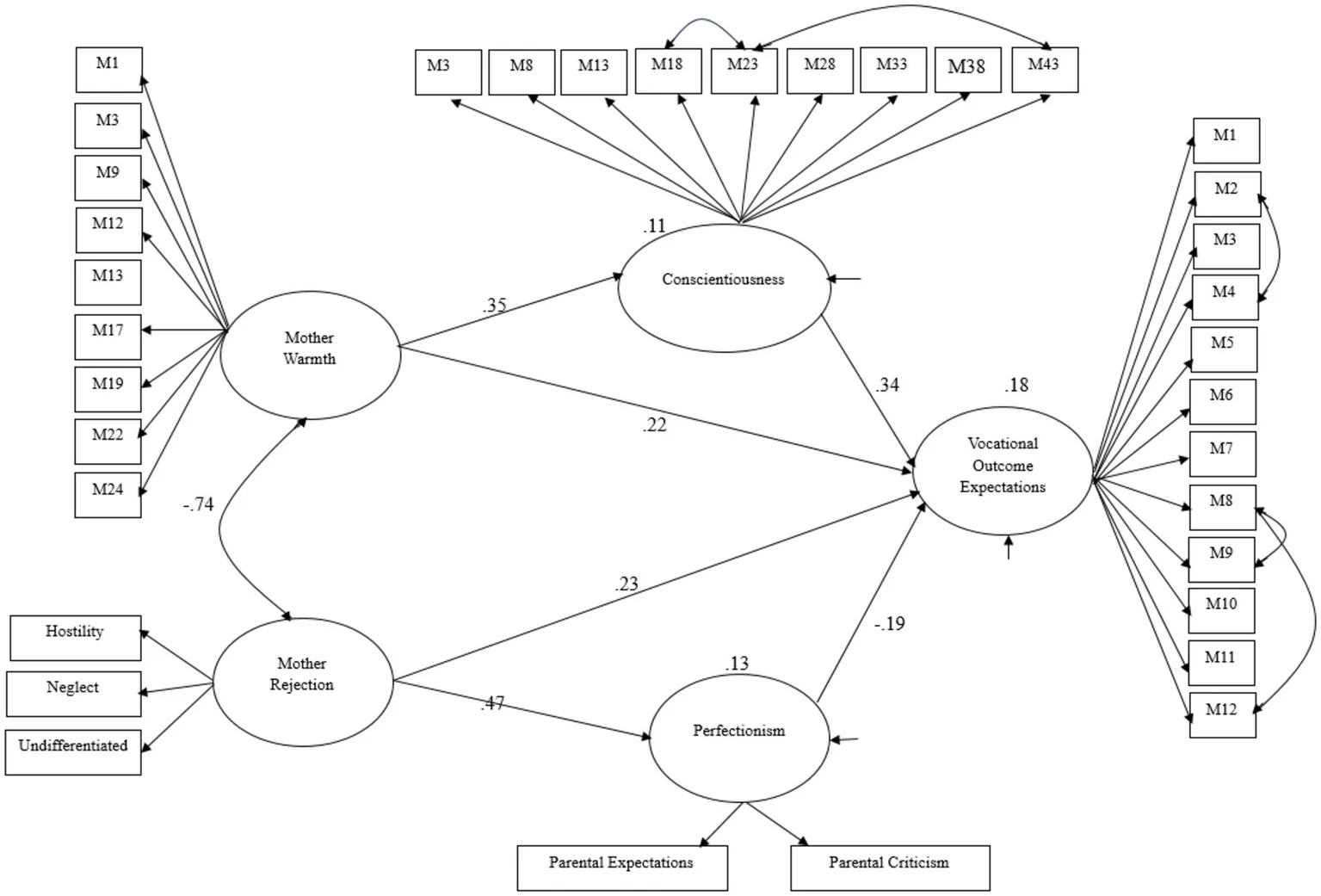
Structural equation modeling analysis of the proposed model of mother acceptance-rejection influences on VOE.

**Figure 2:**
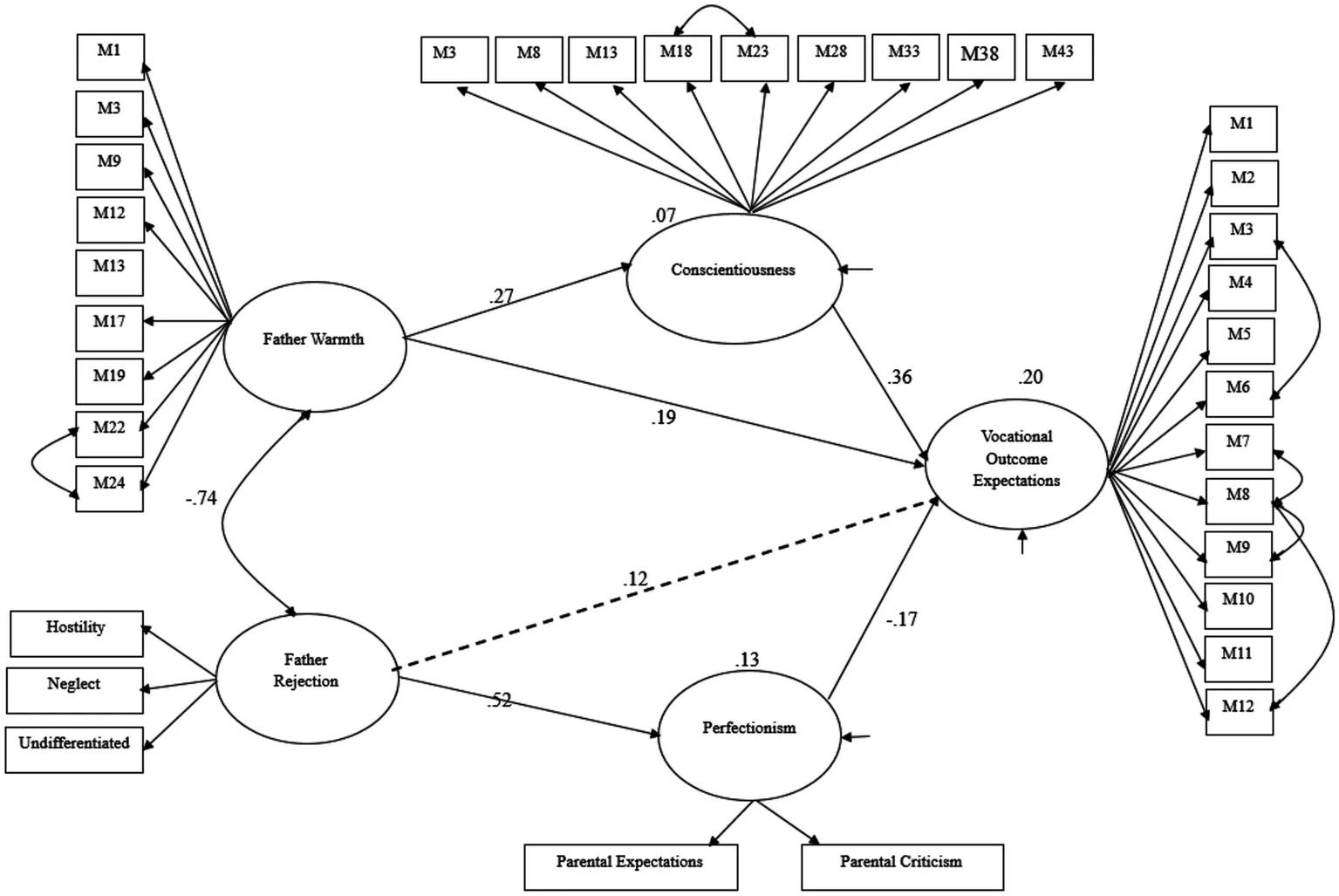
Structural equation modeling analysis of the proposed model of father acceptance-rejection influences on VOE.

After Pearson correlation analyses, the hypothesized models presented in Figures 1, 2 were tested using structural equation modeling (SEM) in IBM SPSS AMOS 28. Model fit was evaluated using the χ^2^/df, RMSEA, CFI, SRMR, TLI, and GFI. The evaluation of model fit was based on the recommendations of Marcoulides and Schumacker (2001) and Tabachnick and Fidell (2001). Accordingly, model fit was considered acceptable when χ^2^/df ≤ 5, RMSEA ≤ 0.10, CFI ≥ 0.90, and GFI ≥ 0.85, SRMR ≤ 0.08, TLI ≥ 0.90 (Beauducel and Wittmann, 2005; Hu and Bentler, 1999; Marcoulides and Schumacker, 2001; Tabachnick and Fidell, 2001). We tested perceived maternal acceptance-rejection and perceived paternal acceptance-rejection in two separate models. The variables perceived maternal warmth, perceived paternal warmth, conscientiousness, and VOE were represented by indicators consisting of items from these variables. Subsequently, we tested structural equation models generated through maximum likelihood estimations to validate the hypothesized models. Finally, to test whether the mediations in the hypothesized models were full or partial, we examined our hypothesized models with and without direct effects of perceived maternal and paternal warmth and rejection on VOE. In this context, we used the Bootstrap method with 5,000 samples to determine the significance of the indirect effects. We examined the significance level of the mediation analysis by considering the 95% confidence interval. Although the measurement instruments utilized different response formats (i.e., 4-point and 5-point Likert scales), no prior standardization or data transformation was applied to the raw scores. In SEM, variables measured on different response scales can be analyzed simultaneously using covariance-based Maximum Likelihood (ML) estimation without requiring prior standardization. To facilitate the interpretation and comparison of effects across constructs with different response formats, completely standardized path coefficients were reported and interpreted.

Because all data were collected using self-report questionnaires, the potential for common method bias (CMB) was assessed using Harman’s single-factor test. All measurement items were entered into an unrotated exploratory factor analysis. The first unrotated factor accounted for 14.92% of the total variance, which is well below the recommended threshold of 50%, suggesting that common method bias is unlikely to represent a substantial threat to the present findings. Furthermore, given the conceptual association between parental warmth and parental rejection, multicollinearity diagnostics were examined for both the maternal and paternal structural models. The variance inflation factor (VIF) values ranged from 1.094 to 3.259, well below the recommended threshold of 5 (Hair et al., 2019), whereas tolerance values ranged from 0.307 to 0.914, indicating no evidence of problematic multicollinearity. These findings indicate that multicollinearity did not pose a threat to the stability of the parameter estimates in either structural model.

## Results

### Preliminary analysis

Tables 1, 2 show the correlations, along with the means and standard deviations, between perceived maternal acceptance/rejection and paternal acceptance/rejection and perfectionism, conscientiousness, and VOE. The results revealed that perceived warmth from both mothers and fathers was positively and significantly correlated with the personality trait of conscientiousness and VOE, and was negatively and significantly correlated with two subdimensions of perfectionism (parental expectations and parental criticism). Furthermore, as expected, the subdimensions of perceived maternal and paternal rejection (hostility, indifference, and undifferentiated rejection) showed positive correlations with the subdimensions of perfectionism (parental expectations and parental criticism). These zero-order correlations were generally aligned with the hypothesized directions of the study’s variables, providing a preliminary foundation for the subsequent structural equation modeling (SEM) analyses.

**Table 1:** Means, standard deviations, zero-order correlations for all variables tested in the paternal model.

Variables	1	2	3	4	5	6	7	8
Father warmth	—							
Father hostility	−0.57**	—						
Father neglect	−0.55**	0.66**	—					
Father undifferentiated	−0.72**	0.79**	0.69**	—				
Conscientiousness	0.21**	−0.20**	−0.20**	−0.20**	—			
Parental expectations	−0.10**	0.26**	0.12**	0.19**	−0.09**	—		
Parental criticism	−0.39**	0.45**	0.35**	0.41**	−0.25**	0.58**	—	
VOE	0.24**	−0.15**	−0.16**	−0.16**	0.27**	−0.02	−0.27**	—
*M*	27.33	8.23	7.89	5.17	32.77	13.62	8.64	37.86
*SD*	6.88	3.05	3.04	2.04	5.00	4.76	3.64	6.39

*p < 0.05, **p < 0.001.

**Table 2:** Means, standard deviations, zero-order correlations for all variables tested in the maternal model.

Variables	1	2	3	4	5	6	7	8
Mother Warmth	—							
Mother Hostility	−0.57**	-						
Mother Neglect	−0.61**	0.62**	—					
Mother Undifferentiated	−0.56**	0.77**	0.71**	—				
Conscientiousness	0.24**	−0.21**	−0.24**	−0.23**	—			
Parental Expectations	−0.15**	0.21**	0.16**	0.15**	−0.09**	—		
Parental Criticism	−0.44**	0.39**	0.33**	0.36**	−0.25**	0.58**	—	
VOE	0.25**	−0.09*	−0.14**	−0.10**	0.27**	−0.02	−0.27**	—
*M*	30.47	8.21	6.55	4.87	32.77	13.62	8.64	37.86
*SD*	5.53	3.00	2.26	1.72	5.00	4.76	3.64	6.39

*p < 0.05, **p < 0.001.

### Control variables and hierarchical regression analyses

To evaluate whether the structural findings were sensitive to the inclusion of demographic covariates, hierarchical regression analyses were conducted separately for the maternal and paternal models. It should be noted that these analyses were employed strictly as a sensitivity check to evaluate the robustness of the main findings, rather than as a substitute for SEM-based covariate adjustment. In the first step, gender and age were entered as control variables, accounting for a negligible proportion of the variance in VOE (*R^2^* = 0.008, *F* (2, 726) = 3.004, *p* = 0.050). In the second step, the primary study variables were entered. For the maternal model, this resulted in a significant increase in explained variance (*ΔR^2^* = 0.159, *ΔF* (7, 719) = 19.676, *p* < 0.001), with conscientiousness (*β* = 0.199, *p* < 0.001), parental expectations (*β* = 0.166, *p* < 0.001), parental criticism (*β* = −0.299, *p* < 0.001), and maternal warmth (*β* = 0.170, *p* < 0.001) remaining significant predictors. Similarly, for the paternal model, the inclusion of the primary study variables resulted in a significant increase in explained variance (*ΔR^2^* = 0.154, *ΔF* (7, 719) = 18.874, *p* < 0.001), with conscientiousness (*β* = 0.193, *p* < 0.001), parental expectations (*β* = 0.160, *p* < 0.001), parental criticism (*β* = −0.289, *p* < 0.001), and paternal warmth (*β* = 0.176, *p* = 0.001) remaining significant. These sensitivity analyses indicate that the structural relationships were not meaningfully altered after controlling for gender and age. Therefore, to preserve degrees of freedom, reduce model complexity, and maintain strict model parsimony, the decision was made to exclude these demographic covariates from the final structural equation models.

### Measurement models

Prior to testing the structural paths, the construct validity and reliability of the measurement models for both maternal and paternal data were evaluated. As shown in Table 3, the composite reliability (CR) values for all constructs exceeded the recommended threshold of 0.70. While the Average Variance Extracted (AVE) values for conscientiousness and VOE were slightly below 0.50, their convergent validity was still established based on Fornell and Larcker's (1981) criteria, given that their CR values were robustly high. Furthermore, all unstandardized factor loadings were significant at the *p* < 0.001 level.

**Table 3:** Standardized factor loadings, composite reliability, and average variance extracted for the measurement models.

Constructs and Items	Standardized Loadings (*λ*)	CR	AVE
Maternal model			
Mother warmth		**0.92**	**0.55**
PARQM items (9 items)	0.67 to 0.80		
Mother rejection		**0.88**	**0.71**
Undifferentiated, neglect, aggression	0.79 to 0.89		
Conscientiousness		**0.72**	**0.25**
BFI items (9 items)	0.21 to 0.71		
Perfectionism		**0.78**	**0.65**
Parental criticism	0.93		
Parental expectations	0.67		
Vocational outcome expectation		**0.91**	**0.45**
VOE items (12 items)	0.42 to 0.81		
Paternal model			
Father warmth		**0.93**	**0.61**
PARQF items (9 items)	0.70 to 0.85		
Father rejection		**0.88**	**0.72**
Undifferentiated, neglect, aggression	0.81 to 0.87		
Conscientiousness		**0.72**	**0.25**
BFI items (9 items)	0.22 to 0.70		
Perfectionism		**0.89**	**0.65**
Parental criticism	0.93		
Parental expectations	0.67		
Vocational outcome expectation		**0.90**	**0.45**
VOE items (12 items)	0.44 to 0.82		

CR = Composite Reliability; AVE = Average Variance Extracted. For brevity, factor loading ranges are presented for multi-item scales. All unstandardized factor loadings were significant at *p* < 0.001.

Although some conscientiousness items exhibited relatively low standardized factor loadings, all original items of the BFI conscientiousness subscale were retained to preserve the theoretical breadth and content validity of the validated instrument rather than removing items solely on statistical grounds. Although the AVE for VOE was slightly below the recommended 0.50 threshold (AVE = 0.45), the construct demonstrated excellent composite reliability (CR = 0.91), and all factor loadings were statistically significant (*p* < 0.001). Consistent with Fornell and Larcker (1981), adequate composite reliability may support the convergent validity of a construct even when the AVE is slightly below the recommended threshold. Furthermore, the Turkish version of the Vocational Outcome Expectations Scale has demonstrated satisfactory validity and reliability (Işık, 2010). Therefore, the construct was retained without modification. To ensure full psychometric transparency, individual item-level standardized factor loadings for all latent constructs across both the maternal and paternal models are provided in Appendix A.

Furthermore, to rigorously establish discriminant validity, the Fornell-Larcker criterion was applied by comparing the square root of the Average Variance Extracted (AVE) for each latent construct against its correlations with all other latent constructs. As shown in the latent correlation matrix (Table 4), the square root of the AVE for each construct is greater than its corresponding off-diagonal correlation coefficients. Notably, discriminant validity between perfectionism and maternal/paternal rejection is firmly established, as the square root of AVE for perfectionism (0.81) exceeds its latent correlations with both maternal rejection (0.443) and paternal rejection (0.506). This confirms robust discriminant validity across all variables in both models.

**Table 4:** Latent correlation matrix and Fornell-Larcker criterion for discriminant validity.

Variables	1	2	3	4	5
1. Parental warmth	**0.74/0.78**	−0.727	0.267	−0.406	0.262
2. Parental rejection	−0.718	**0.84/0.85**	−0.261	0.506	−0.183
3. Conscientiousness	0.322	−0.308	**0.50/0.50**	−0.273	0.384
4. Perfectionism	−0.467	0.443	−0.272	**0.81/0.81**	−0.265
5. Vocational outcome expectaiton	0.252	−0.116	0.385	−0.265	**0.67/0.67**

The lower diagonal represents the latent correlations for the Maternal Model, and the upper diagonal represents the latent correlations for the paternal model. The bolded values on the diagonal represent the square roots of the Average Variance Extracted (AVE) for the Maternal/Paternal models, respectively. All correlations are statistically significant at *p* < 0.01.

Instead of constructing our models based on parental acceptance and rejection, we constructed them separately for perceived acceptance and rejection from mothers and fathers. We wanted to reveal the differences between the effects of perceived acceptance and rejection from mothers and fathers on VOE. The initial test results of the measurement models constructed for mothers and fathers did not demonstrate adequate fit. Therefore, the model was modified, and correlations were generated among the six error terms in the model. After modification, the measurement model improved significantly and showed good fit (Table 5). The factor loadings of all latent variable indicators were also significant (*p* < 0.001) (Table 6).

**Table 5:** Model fit indices for measurement models and structural models.

Model	χ2/df (χ2, df)	RMSEA [90% CI]	SRMR	CFI	TLI	GFI
Maternal model	2.79 (1536.76, 549)	0.05 [0.047, 0.053]	0.05	0.915	0.907	0.89
Paternal model	2.94 (1613.74, 548)	0.052 [0.049, 0.055]	0.05	0.916	0.909	0.885

**Table 6:** Standardized estimates and 95% CIs for indirect influences of parental acceptance-rejection on vocational outcome expectation via personality factors.

Paths	Total effect *(β)*	Direct effect *(β)*	Direct effect *p-*value and 95% CI	Indirect effect *(β)*	Indirect effect *p*-value and 95% CI	*SE*	Mediation type
Maternal model							
Warmth → Consc. → VOE	0.34	0.22	*p* < 0.05, [0.09, 0.35]	0.12	p < 0.05, [0.08, 0.17]	0.02	Partial Mediation
Rejection → Perf. → VOE	0.14	0.23	*p* < 0.05, [0.11, 0.36]	−0.09	p < 0.05, [−0.14, −0.05]	0.02	Inconsistent Mediation (Suppression)
Paternal model							
Warmth → Consc. → VOE	0.29	0.19	*p* = 0.050, [0.06, 0.31]	0.10	p < 0.05, [0.06, 0.14]	0.02	Partial Mediation
Rejection → Perf. → VOE	0.03	0.12	*p* = 0.813, [−0.15, 0.26]	−0.09	p < 0.05, [−0.15, −0.04]	0.02	Full Mediation

During the evaluation of the measurement models, specific error terms were allowed to correlate based on modification indices to improve model fit. It is widely acknowledged that correlating error terms purely based on data-driven indices can increase the risk of model overfitting and capitalization on chance (MacCallum et al., 1992). To avoid this, error covariances were only specified between items loading on the same latent construct. For instance, correlated errors were added within the Conscientiousness subscale (e.g., items M18 and M23), the Vocational Outcome Expectations subscale (e.g., items M8 and M9; M8 and M12), and the Parental Warmth subscales (e.g., M22 and M24). Therefore, these covariances are theoretically justified because they capture shared residual variance arising from similar item wording or overlapping item content within the same latent construct, rather than reflecting arbitrary associations across different constructs.

### Structural models

After clarifying the measurement models, we tested the four hypotheses with a structural model (Figures 1, 2). Hypothesis 1 posited that perceived parental warmth (H1a maternal warmth, H1b paternal warmth) had direct and indirect relationships with VOE. To test this, we examined the potential mediation of conscientiousness between perceived maternal/paternal warmth and VOE. Hypothesis 2 posited that parental rejection (H2a maternal rejection, H2b paternal rejection) significantly and negatively influences VOE. Additionally, Hypotheses 3 and 4 tested whether conscientiousness mediated the effect of parental acceptance on VOE, and whether perfectionism mediated the effect of parental rejection on VOE, respectively. In the mediation analysis conducted with SPSS AMOS, the highest and lowest values of the confidence intervals were used to determine whether the mediation relationship was significant. Mediation is considered significant if the bootstrap confidence interval does not include zero (Hayes, 2017). In this context, we used the Bootstrap method with 5,000 samples and examined the significance level of the mediation analysis considering the 95% confidence interval. The significance level was determined as *p* < 0.05.

#### The maternal model

In the maternal model, the paths from perceived maternal warmth to conscientiousness (*β* = 0.35) and VOE (*β* = 0.22) were significant (*p* < 0.05). When we included conscientiousness in the analysis, the direct effect of perceived maternal warmth on VOE decreased, but the effect remained statistically significant, indicating that conscientiousness partially mediated the relationship. Thus, Hypotheses 1a and 3a were fully supported. Furthermore, the paths from perceived maternal rejection to perfectionism (*β* = 0.47) and VOE (*β* = 0.23) were significant (*p* < 0.05). When we included perfectionism in the analysis, the direct effect of perceived maternal rejection on VOE decreased, but the effect remained statistically significant (Table 5). Although the bivariate correlations indicated that maternal rejection dimensions-hostility (*r* = −0.09, *p* < 0.05), neglect (*r* = −0.14, *p* < 0.001), and undifferentiated rejection (*r* = −0.10, *p* < 0.001) were negatively associated with VOE, the direct structural path from maternal rejection to VOE was found to be positive and significant (*β* = 0.23, *p* < 0.05). This change in direction represents a classic suppression effect. In the structural model, maternal warmth and maternal rejection share a very high amount of variance (with a correlation of −0.74 between the latent constructs). When maternal warmth and the mediators (conscientiousness and perfectionism) partition out this shared variance, the remaining unique variance of maternal rejection predicts VOE positively. Because this direct path did not emerge in the hypothesized negative direction, Hypothesis 2a was not supported. However, the mediation hypothesis H4a was fully supported, showing that perfectionism successfully mediated the relationship.

#### The paternal model

Similarly, the second model tested the relationships for perceived paternal warmth and rejection. The paths from perceived paternal warmth to conscientiousness (*β* = 0.27) and VOE (*β* = 0.19) were significant (*p* < 0.05). When we included conscientiousness in the analysis, the direct effect of perceived paternal warmth on VOE decreased, but the effect remained statistically significant. Conscientiousness partially mediated the relationship, fully supporting Hypotheses 1b and 3b. Furthermore, the path from perceived paternal rejection to perfectionism (*β* = 0.52, *p* < 0.05) was significant, whereas the direct path from perceived paternal rejection to VOE was not significant (*β* = 0.12, *p* = 0.813). Consequently, Hypothesis 2b was not supported as a direct effect. Rather than interpreting this pattern as traditional full mediation, perceived paternal rejection was indirectly associated with VOE through perfectionism, while the direct path remained non-significant. Thus, the indirect association proposed in Hypothesis 4b was supported.

## Discussion

In this study, we sought to understand how positive and negative environmental factors are associated with university students’ future visions through personality traits. To this end, we examined the mediating role of the personality traits of conscientiousness and perfectionism in the relationship between parental acceptance-rejection and VOE. The results confirmed the hypothesis that parental acceptance is positively associated with VOE (H1), with the personality trait of conscientiousness is the mediator (H3). These results support the SCCT’s view of the role of parents in career development. Accepting parents’ attitudes and behaviors provides individuals with a supportive environment, which can increase their motivation and self-confidence (Casino-García et al., 2021). Rohner et al. (2012) suggest that the need for positive response reflects the human tendency to seek care, attention, support, and affection, and that this need becomes more complex in adulthood. When this need is unmet, individuals tend to exhibit anxious and insecure behavior. Overall, the study demonstrated the positive relationship of an accepting, warm family environment on an individual’s VOE. In a family environment with unconditional acceptance, individuals may be able to develop achievable goals, realistic plans, and self-discipline and organization to achieve these goals. Parental attitudes that balance support and expectations help university students develop academic resilience (Morales-Rodríguez, 2021). It appears that students perceive themselves as more successful in coping with academic obstacles when they have strong support. Consequently, they might approach their future more optimistically, and their expectations regarding their professions may be more positive.

While this study did not support our hypotheses regarding a direct negative association between parental rejection and VOE (H2), it revealed that parental rejection had an indirect effect on VOE through the mediating role of perfectionism (H4). Although the bivariate correlations results align with previous findings indicating that perceived maternal rejection is negatively associated with VOE in a Turkish sample (Özden, 2014) and that perceived paternal acceptance predicts occupational and academic adjustment in a Chinese sample (Niu et al., 2024), our structural model revealed a different pattern of associations. Specifically, perfectionism acted as an inconsistent mediator (suppression effect) in the relationship between maternal rejection and VOE, whereas perceived paternal rejection was indirectly associated with VOE through perfectionism, while the direct path was not statistically significant. The positive direct association between maternal rejection and VOE warrants careful theoretical consideration within the context of a suppression effect. When the shared variance of maternal warmth is statistically partitioned out, the unique variance of maternal rejection may reflect a compensatory motivational process in some emerging adults. Particularly when the negative psychological burden of perfectionism is controlled, maternal rejection may, under certain conditions, be associated with higher VOE by motivating university students to seek greater autonomy, self-reliance, and financial independence. Perfectionistic individuals typically set exceedingly high standards for themselves and exhibit heightened sensitivity to meeting these standards. According to IPARTheory, experiences of parental rejection activate stress responses and influence emotional regulation systems (Rohner and Lansford, 2017). Children exposed to high parental expectations and criticism may come to perceive themselves as valued only when they meet these expectations and perform flawlessly. Accordingly, parental rejection (Yakın, 2011), high parental expectations, and harsh criticism have been linked to perfectionism in young people (Curran and Hill, 2022; Smith et al., 2022). Individuals who perceive greater parental rejection may adopt excessively high standards in an effort to gain parental acceptance, and the anxiety and self-doubt associated with striving for these standards may undermine their VOE. Nevertheless, this interpretation should be considered tentative. Given the suppression (inconsistent mediation) pattern observed in the present study, the positive direct association between maternal rejection and VOE should not be interpreted as a stable developmental conclusion. Future longitudinal studies are needed to replicate this finding and clarify the underlying mechanisms.

Furthermore, the results suggest that the conscientiousness personality trait serves as a key underlying mechanism (mediator) that facilitates this positive association. Conversely, perceived parental rejection was found to have an indirect negative association with VOE through the mediating role of perfectionism, while perfectionism itself directly and negatively predicted VOE. While the results provide insight into the association between parental acceptance-rejection and future expectations through personality traits, the study was conducted with university students. VOE is open to change over time and is crucial in revealing an individual’s self-perceptions, attitudes, goals, and interests (Işık, 2010). Therefore, we should note that this was a cross-sectional study conducted with participants whose average age was 21 and who were all still in education. It should be reexamined with longitudinal studies. Furthermore, the study suffers from all the limitations of self-report measures and online data collection. Although Harman’s single-factor test suggested that common method bias was not a major issue, the reliance on data collected simultaneously from the same participants means that this bias cannot be completely ruled out. Therefore, we recommend using more diverse assessment methods, particularly on a sensitive topic such as parental rejection, that allow for more comprehensive and collaborative interviews with participants. Another methodological limitation concerns the measurement properties of the study instruments. Although the conscientiousness construct demonstrated acceptable composite reliability in both models (CR = 0.72), its AVE was below the recommended threshold, indicating limited convergent validity. Therefore, findings involving conscientiousness should be interpreted with some caution. Future research may examine the construct using alternative measures or revised item sets to strengthen convergent validity. Additionally, the characteristics of our sample present important limitations regarding generalizability. The data were collected from a single large university in the Marmara Region of Turkey and exhibited a significant gender imbalance, comprising 610 female and 119 male students. Consequently, the findings of the present study may not generalize to university students from other Turkish regions, individuals attending different types of educational institutions, more gender-balanced populations, or emerging adults who are not enrolled in higher education.

## Data Availability

The datasets presented in this article are not readily available because the data obtained in our study have been restricted to the project team by the ethics committee in order to protect participant confidentiality. Therefore, making the data publicly available would not be consistent with ethical principles. The findings of our study are presented in detail within the article. Requests to access the datasets should be directed to gultekinfiliz@uludag.edu.tr.
