# Supplementary material for: Parental warmth, parental rejection, and vocational outcome expectations: the mediating roles of conscientiousness and perfectionism
Source: Front Psychol. 2026 Aug 7;17:1861992. doi: 10.3389/fpsyg.2026.1861992 (PMC13493379; doi:10.3389/fpsyg.2026.1861992)
Supplement: Supplementary file 1 [file Table_1.pdf]

Appendix A

Table A1. *Item-Level Standardized Factor Loadings for the Maternal and Paternal Models*

Latent Construct and Indicators/Items	Maternal Model (λ)	Paternal Model (λ)
**Parental Warmth (Anne/Baba Kabul)**		
PARQM1 / PARQF1	.74	.77
PARQM3 / PARQF3	.72	.75
PARQM9 / PARQF9	.77	.83
PARQM12 / PARQF12	.67	.73
PARQM13 / PARQF13	.78	.85
PARQM17 / PARQF17	.80	.83
PARQM19 / PARQF19	.76	.82
PARQM22 / PARQF22	.69	.70
PARQM24 / PARQF24	.71	.76
**Parental Rejection**		
Undifferentiated Rejection	.89	.87
Neglect/Indifference	.79	.81
Hostility/Aggression	.84	.87
**Conscientiousness**		
BFI3	.71	.70
BFI8	**.21**	**.22**
BFI13	.54	.52
BFI18	.30	.30
BFI23	.48	.50
BFI28	.48	.47
BFI33	.69	.68
BFI38	.54	.55
BFI43	**.25**	**.29**
**Perfectionism**		
Parental Criticism	.92	.93
Parental Expectations	.67	.67
**Vocational Outcome Expectations (VOE)**		
VOE1	.65	.66

VOE2	.66	.69
VOE3	.74	.71
VOE4	.65	.67
VOE5	.57	.58
VOE6	.78	.76
VOE7	.74	.72
VOE8	.67	.64
VOE9	.60	.60
VOE10	.82	.82
VOE11	**.43**	**.45**
VOE12	.68	.68

Note. All factor loadings are statistically significant at p<.001. Bold values represent the lower factor loadings discussed as measurement limitations in the text.
